# Calibration of an intrahost malaria model and parameter ensemble evaluation of a pre-erythrocytic vaccine

**DOI:** 10.1186/1475-2875-14-6

**Published:** 2015-01-07

**Authors:** Kevin A McCarthy, Edward A Wenger, Grace H Huynh, Philip A Eckhoff

**Affiliations:** Institute for Disease Modeling, 1555 132nd Ave NE, Bellevue, WA 98005 USA

**Keywords:** Pre-erythrocytic, Calibration, Ensemble, Vaccine, Model

## Abstract

**Background:**

A pre-erythrocytic vaccine could provide a useful tool for burden reduction and eventual eradication of malaria. Mathematical malaria models provide a mechanism for evaluating the effective burden reduction across a range of transmission conditions where such a vaccine might be deployed.

**Methods:**

The EMOD model is an individual-based model of malaria transmission dynamics, including vector lifecycles and species-specific behaviour, coupled to a mechanistic intrahost model of malaria parasite and host immune system dynamics. The present work describes the extension of the EMOD model to include diagnoses of severe malaria and iterative calibration of the immune system parameters and parasite antigenic variation to age-stratified prevalence, incidence and severe disease incidence data obtained from multiple regions with broadly varying transmission conditions in Africa. An ensemble of calibrated model parameter sets is then employed to evaluate the potential impact of routine immunization with a pre-erythrocytic vaccine.

**Results:**

The reduction in severe malaria burden exhibits a broad peak at moderate transmission conditions. Under sufficiently intense transmission, a vaccine that reduces but does not eliminate the probability of acquisition from a single challenge bite may delay infections but produces minimal or no net reduction. Conversely, under sufficiently weak transmission conditions, a vaccine can provide a high fractional reduction but avert a relatively low absolute number of cases due to low baseline burden.

**Conclusions:**

Roll-out of routine immunization with pre-erythrocytic malaria vaccines can provide substantial burden reduction across a range of transmission conditions typical to many regions in Africa.

**Electronic supplementary material:**

The online version of this article (doi:10.1186/1475-2875-14-6) contains supplementary material, which is available to authorized users.

## Background

Recent years have seen encouraging progress in the reduction of global malaria burden, with the WHO’s global mortality estimates declining roughly 30% from 2000 to 2013 [1]. However, malaria continues to kill hundreds of thousands a year, with the burden of mortality falling most heavily on children in sub-Saharan Africa. Improvements in vector control, diagnostics and treatment have all contributed to the declining mortality [2–4], and a preventative vaccine could provide an important additional tool to the malaria control and eradication effort. Mathematical models of malaria transmission and within-host disease progression can aid in understanding the effects of potential vaccine distributions under a broad range of transmission conditions. A variety of potential vaccines are under investigation; depending on which stage of the parasite lifecycle is targeted, a vaccine could act to prevent human acquisition, reduce morbidity post-acquisition, or prevent transmission from human hosts back to the mosquito vectors.

The EMOD model is an individual-based, stochastic simulation framework that couples a mechanistic model of intra-host parasite lifecycle and immune system response dynamics to a detailed model of the mosquito population lifecycle, including responses to changes in climate and descriptions of species-dependent behaviours [5–9]. Campaign interventions targeting the vector population (insecticide-treated nets, habitat clearance) or the human population (vaccines, anti-malarial drug regimens, etc.) can be flexibly distributed to specified subpopulations, e.g., age-based immunization schedules, calendar-based distribution campaigns or treatment-seeking upon onset of symptoms.

Malaria control efforts rely on multiple tools to reduce disease burden: vector control techniques, chemoprophylaxis, and post-exposure drug treatment [10]. The proliferation of drug-resistant parasites [11] or insecticide-resistant mosquitos [12] remains a concern for these techniques, and malaria vaccines could provide a crucial tool in burden reduction and potential elimination of malaria. Pre-erythrocytic vaccines target the *Plasmodium falciparum* parasite prior to blood-stage disease, aiming to block liver-stage infection from occurring or from progressing to blood-stage infection, thus preventing the onset of symptomatic malaria as well as onward transmission of gametocytes. Among current pre-erythrocytic vaccine candidates, the RTS,S vaccine is in the most advanced stage of clinical trials [13–15]. Human trials of the vaccine (with various adjuvants) have been conducted since the early 1990s, demonstrating safety and immunogenicity in clinical and field settings [16]. A recent Phase 3 trial across eleven African sites [13] reports promising results; a 3-dose infant immunization schedule provides efficacies against clinical malaria of 46% over an 18 month follow-up period, with similarly strong efficacies against severe malaria and malaria hospitalization (34% and 41%, respectively).

An effective pre-erythrocytic vaccine could provide a useful tool for burden reduction and eventual eradication of malaria. However, a vaccine that provides limited protection against infection could delay the development of adaptive immunity to blood-stage disease, increasing the malaria burden in older children whose vaccine-derived immunity has waned. This “rebound effect” has been observed to occur in field studies of intermittent preventive treatment and chemoprophylaxis [17, 18], and should be investigated in the case of pre-erythrocytic vaccines. Evaluation of the effective burden reduction across the broad range of transmission conditions where such a vaccine could be deployed is a natural target for mathematical malaria models [19, 20].

## Methods

The EMOD malaria model has been described in detail in previous papers [5–9]. The intrahost model features a mechanistic description of within-host infection dynamics that lead to the acquisition of parasitological and clinical immunities with repeated exposure. This model includes descriptions of both the innate and adaptive human immune responses to antigens presented by merozoite surface proteins (MSP), unique epitopes of *P. falciparum* erythrocyte membrane proteins (PfEMP), and cross-reactive PfEMP epitopes. Each of these antigenic compartments is also allowed a configurable number of antigenic variants [8, 21–23]. The adaptive immune system response to each variant within each antigenic compartment is described by a growth rate, capacity, decay rate, memory level, and parasite clearance efficacy. The unique PfEMP epitopes are also characterized by a switching rate, allowing the presentation of distinct variants over the course of a single infection. Individual body temperatures and red blood cell (RBC) concentrations are tracked throughout the simulation to identify clinical and severe disease incidence (defined in Calibration subsection). Body temperature is governed by innate cytokine production, which is stimulated by parasite density (through a pyrogenic threshold) and schizont rupture events, and mitigated by the production of adaptive antibodies [6]. RBC concentrations are governed by processes of destruction through schizont rupture and production by the host.

### Calibration

Prior to evaluating the efficacy of a pre-erythrocytic vaccine in field conditions, the model was calibrated in two steps. First, age-stratified prevalence and fever incidence curves were used to calibrate parameters governing antigenic dynamics and immune response, and then age-stratified severe disease incidence curves were used to calibrate model parameters governing the probabilistic presentation of a severe malarial incident.

The parameters of the immune system model that are most relevant to acquisition of long-term clinical and parasitological immunity were calibrated to age-stratified prevalence and incidence data from six sites experiencing measured entomological inoculation rates (EIRs) ranging from 18 to over 300. Prevalence data comes from Matsari, Sugungum and Rafin Marke in Nigeria [24], and Namawala, Tanzania [25]; incidence data was obtained from Ndiop and Dielmo, Senegal [26–28].

Clinical incidence data present some challenges for model calibration. In a model of malaria transmission with no other pathogens, as is the case here, all fevers are caused by malaria. This is not the case in field studies, and the attribution of clinical symptoms can be problematic, particularly at high prevalence. Studies often use cutoffs on the parasitaemia level concurrent with fever incidence to determine a ‘malaria attributable fraction’ , but this is subject to heterogeneities based on local transmission intensity [29]. Other works have also noted that higher frequencies of active case detection visits tend to produce higher estimates of incidence [30, 31]. It is not obvious whether this correlation arises because of over-attribution of non-malarial fevers caught in frequent visits, or to true malaria-attributable fevers being missed in between visits. At these Senegal sites, patients were visited three times per week, and the present work makes no attempt to ‘correct’ the data for case detection frequency or to censor model-predicted fevers from being attributed to malaria if concurrent parasitaemia is low. Such corrections may become necessary when expanding the set of calibration sites or comparing predicted incidence rates against out-of-sample data.

At the Senegal study sites, patients were also treated with quinine when fever presented with concurrent parasitaemia. The high rate of case detection combined with rapid treatment could exert strong effects on the development of immunity. To account for this, the simulated population undergoes a burn-in of forty years, during which the population is exposed to the expected transmission conditions but does not undergo rapid case detection and treatment; this setup prevents high treatment rates from limiting age-dependent immunity development in the population. After the burn-in period, fever incidence reporting begins, and individuals who present fever begin parasite-clearing treatment with a 30% probability per day from the fever onset, meant to roughly capture the ‘time to next visit’ for fever detection. The rapid parasite clearance after fever onset also likely prevents the dataset from informing the model calibration with respect to the effects of coinfection on morbidity.

The simulated transmission conditions at each site are characterized by a total EIR and a normalized seasonal profile. Simulations with local climate data were necessary to determine the seasonal profile of certain sites, while others were taken from literature. Throughout this study, the annual EIR characterizes the biting rate experienced by grown adults; children experience a reduction in this biting rate described by a piecewise-linear function that approximates their expected surface area as a function of age (i.e., biting rates are assumed to be proportional to skin surface area). Age-dependent biting is well documented [32, 33] and has been used in previous modelling work [34].

Because of the interactions between the human and vector systems in the EMOD model, broad variations in human immune dynamics induce variations in the transmission conditions. This presents a difficulty in the calibration process, as the target distributions are age-specific prevalence and incidence curves determined at measured EIRs. It is computationally expensive to adjust vector habitats at each tested set of intrahost parameters to compare results at equal transmission levels. To remove this complication, the measured transmission conditions were reproduced by removing the vectors from the simulation entirely, and instead subjecting the simulated individuals to periodic sporozoite challenge at a known intensity. The frequency of challenge is varied monthly to reproduce seasonal variation in EIR.

The model parameters included in the calibration are the number of antigenic variants in each of the three immune compartments, the killing strengths of the MSP antigens and shared minor epitopes, and the PfEMP antigenic switching rate; other parameters of the model had been calibrated to reproduce individual time courses of infection and distributions of infection durations and were held fixed during this calibration.

For each set of model input parameters, a set of simulations are run to characterize outputs under the measured transmission conditions at each of the field sites from the data, and the overall likelihood of an input parameter set is the product of likelihoods over the six sites. Simulated prevalence output is modelled as random binomial slide positivity measurements, characterized by a number of slides viewed and an individual’s current blood parasite density. The definition of a clinical malarial incident is configurable in the EMOD model; for this study, a clinical incident begins when an individual’s body temperature is raised by 1.5°C. Parasite density alone does not trigger a clinical incident, though temperature and parasite density are implicitly linked through the innate immune response. The clinical incident continues until the fever remains below 0.5°C for two weeks; this refractory period prevents the multiple recrudescent fever events typical to malaria from being recorded as multiple independent clinical incidents.

Incremental mixture importance sampling [35, 36] was employed for parameter space exploration and calibration. Once the target posterior likelihood has been sufficiently well-sampled, this stage of calibration is finished by producing an acceptable region of parameter space, defined by placing a threshold on the overall log-likelihood (based on Wilks’ theorem) and forming the convex hull of all parameter sets above this threshold. This calibrated parameter volume is used as an acceptance region for resampling model parameter sets. More details of the simulation setup, likelihood functions and algorithm can be found in Additional file 1.

**Figure 1:**
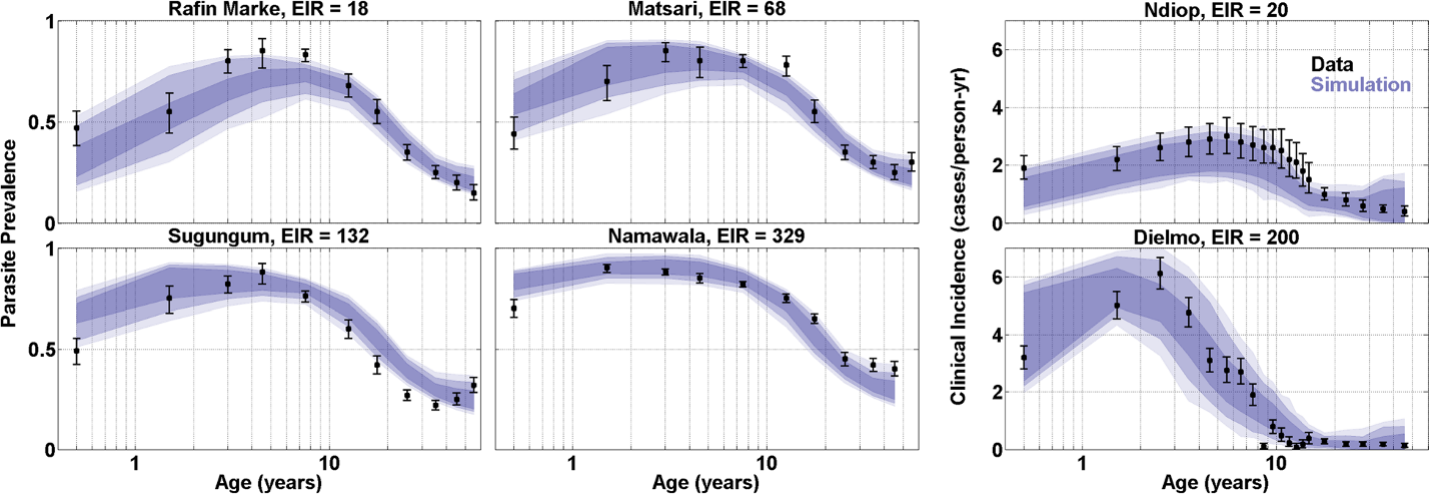
**(Two left columns) Age-stratified prevalence data and simulation results from four regions in Nigeria and Tanzania.** (Right column) Age-stratified incidence data and simulation results from two regions in Senegal. In both panels, the blue-shaded regions represent the 68/95/100% quantiles of simulations passing the likelihood threshold.

The results of the model calibration to age-stratified measurements of parasite prevalence and clinical incidence are presented in Figure 1. The two left columns present the measured fraction of children testing positive for parasites by slide microscopy at four sites with annual EIRs ranging from 18–329. The data are presented as black points with statistical error bars, and the blue-shaded regions that enclose the results of all simulations passing the total log-likelihood threshold (100/95/68% quantiles are presented in progressively darker shading). The right column similarly presents the rate of fever incidence at two sites, with annual EIRs of 20 and 200.

Sites with increasing annual EIR exhibit a more rapid rise in detectable parasitaemia levels at the youngest ages and a higher overall peak prevalence, but all sites exhibit a decrease in prevalence over the second decade of life. Above 20 years of age, the observed prevalence does not vary dramatically over this order-of-magnitude range of transmission intensities. In the two Senegal sites, an order of magnitude increase in EIR produces a substantially higher peak rate of clinical incidence, an earlier peak age (2–3 in Dielmo *vs* 5–10 in Ndiop), and a lower incidence rate in adults (approximately 0.6/year in Ndiop *vs* 0.2/year in Dielmo). The more intense transmission in Dielmo confers sufficient immune protection that the cumulative lifetime incidence is lower than in Ndiop [26].

The effects of each of the calibration parameters on the features described above are not simple to describe, as there are strong interactions among the terms. Broadly, increasing the number of antigenic variants in the three immune compartments delays the onset of parasitological and symptomatic immunity, while the antibody killing strengths to MSP and cross-reactive PfEMP epitopes most strongly affect the magnitude of the prevalence and incidence in early ages. The antigenic switching rate also strongly affects the onset of symptomatic immunity; when the switching rate is too slow, immunity is acquired slowly as the host is presented with few variants in any individual infection. The antigenic switching rate has also been constrained by previous work on the duration of infection and infectiousness in naïve individuals [8].

The calibration procedure continues by sampling parameter values from the acceptable regions defined in the first stage and varying a new set of parameters governing severe disease, targeting data on age-specific severe disease rates at five sites in The Gambia and Kenya [37], and proportional rates of cerebral or anemic malaria from seventeen sites across Africa [38]. In the EMOD model, an individual’s current state is mapped onto three probabilities of diagnosis of severe malaria due to three underlying causes. An individual’s current RBC count sets a probability of diagnosis due to anaemia and associated presentations, their current body temperature acts as a proxy for presentation of severe cerebral malaria, and their current parasitaemia level acts as a catch-all proxy for other complications (e.g., respiratory involvement). Similar protections to those described above regarding clinical incidents are in place to prevent a single severe presentation from being recorded as multiple incidents.

The functions translating RBC counts, asexual parasite densities and fever levels to probabilities of diagnosis are sigmoid (logistic) functions characterized by a width and a midpoint. The functional form is:


Where *k* represents the inverse width, and *x*_*0*_ is the threshold, at which the probability is equal to 0.5. The calibration includes the widths and midpoints associated with severe cerebral malaria or severe parasitaemia, a multiplier governing how many red blood cells are destroyed when a schizont ruptures, and the level of maternal antibody protection (which varies from site to site based on measured transmission conditions – see “Details of calibration setup” in Additional file 1 for more details). The width and threshold for severe anaemia is not included in the calibration, because this diagnosis in the source data is based on a well-defined threshold at haemoglobin density of 5 g/dl. The frequency of anaemia is governed by the competing processes of erythrocyte destruction by rupturing schizonts and increased erythropoiesis in response to decreased haemoglobin density. Because these two effects produce opposing results on the rate of severe anaemia, the parameter governing erythropoiesis was fixed to a value hand-calibrated to measured individual time courses in naïve patients, and only the erythrocyte destruction factor is varied in this calibration.

**Figure 2:**
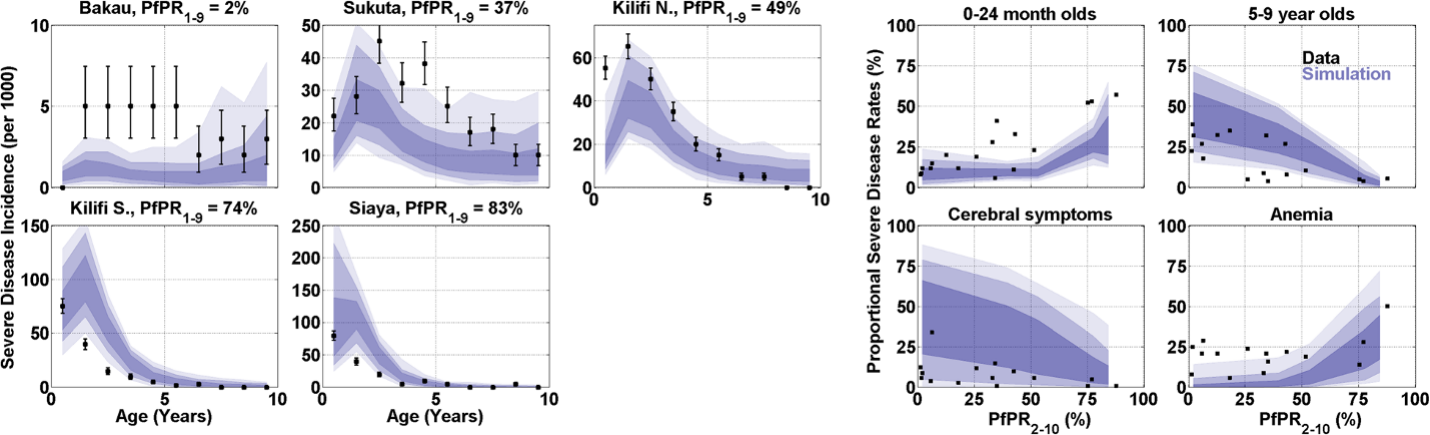
**(Left) Age-stratified severe disease incidence data and results from simulations from five regions in Africa; note the changing y-axis when comparing.** (Right) Proportions of severe disease incidence in various categories. In both panels, the blue-shaded regions represent the 68/95/100% quantiles of simulations passing the likelihood threshold. PfPR_2–10_ is the proportion of 2–10 years olds testing positive for falciparum parasites.

The results of calibrating the model to age-stratified severe disease diagnoses are presented in Figure 2. Age-stratified total severe disease incidence rates, measured from hospital records and census data, at five sites with parasite prevalence rates in one to nine years olds ranging from 2 to 83% (mapped through simulation to annual EIRs ranging from 0.1 to 55) are presented on the left. The proportions of severe disease incidence in the under 24 months and five to nine years age bins, and the proportions of cerebral and anemic disease at 17 sites, along with simulation results, are presented on the right. The blue-shaded regions are defined as in Figure 1.

In the parameter region favored post-calibration, the probability of experiencing a severe cerebral malaria episode is essentially zero for low-grade fevers and increases rapidly over a range of body temperatures from 40-41°C (base body temperature is assumed to be 37°C). Similarly, a severe disease episode caused by hyperparasitaemia is highly unlikely at parasite densities below approximately 200,000 per μl, rapidly increasing to a probability approaching 1 by approximately 400,000 per μl (figures available in Additional file 1).

Sites with increasing transmission intensity exhibit an earlier peak age in severe disease rate, and a corresponding increase in the proportion of severe disease due to anemia. Maternal antibodies play a large role in the initial rise in severe incidence. The degree of protection conferred by maternal antibodies was modelled as an increasing function of transmission intensity, but a mother’s antibody levels against particular antigenic variants were not considered in these simulations. The cumulative severe disease incidence is highest at moderate EIRs. The model predicts that under intense transmission conditions, many children are exposed to the parasite while maternal antibodies still provide sufficient protection to prevent severe symptoms, and these early exposures confer a degree of protection against severe malaria in future infections. This idea has also been hypothesized to explain observations that cumulative paediatric severe malaria incidence is highest under moderate conditions and declines at extremely high levels of transmission [37, 39]. The other five calibration parameters interact strongly in setting the overall rate of severe disease incidence as well as the proportions of severe disease ascribed to the three underlying mechanisms.

The limitations of this calibration procedure are worth addressing here. The prevalence and incidence calibration was performed only to sites experiencing annual EIRs > 18, which represents substantially higher transmission intensity than currently experienced by most of the African population [40]. To provide confidence that the calibrated model can reasonably be applied to study lower transmission intensities, the results of this calibration procedure were checked against data outside of the calibration sample down to annual EIRs of 1. Details and figures are contained in Additional file 1. Individuals in the model are also homogenous in terms of immune system dynamics, response to treatment, and biting risk. Individual- and site-level heterogeneities in these quantities and others have been observed in studies [14, 41–45] and can exhibit significant effects on population-level data. These effects are under consideration in extensions to the present work.

### Parameter ensemble model vaccine evaluation

After calibration, the effects of a potential rollout of a pre-erythrocytic vaccine were evaluated across a wide range of transmission conditions and for an ensemble of acceptable input parameters. Each parameter set in the ensemble represents an independent set of input parameters sampled from the 12-dimensional calibrated parameter space. One-hundred and seventy parameter sets were included in the ensemble; this number resulted from available computation time, rather than any considerations specific to the analysis. Each of the 170 models was run under a variety of transmission conditions; these were not explicitly based on particular locations, but on a set of four seasonal profiles and 18 total annual EIR magnitudes from 1 to 300. The four seasonal profiles included two realistic profiles based on Sugungum, Nigeria and Mocuba, Mozambique; a non-seasonal profile (constant rate of biting year-round); and a highly seasonal profile in which all biting takes place at a flat rate for six months and no biting occurs for the remaining six months. Eight stochastic repetitions of each model/transmission scenario were performed, beginning at different times of the year to ameliorate potential systematic transient effects. Finally, each setup was run once without vaccine distribution to provide baseline incidence rates, and run twice more with different vaccine efficacy profiles. Simulation outputs are binned into six-month time periods for analysis.

**Figure 3:**
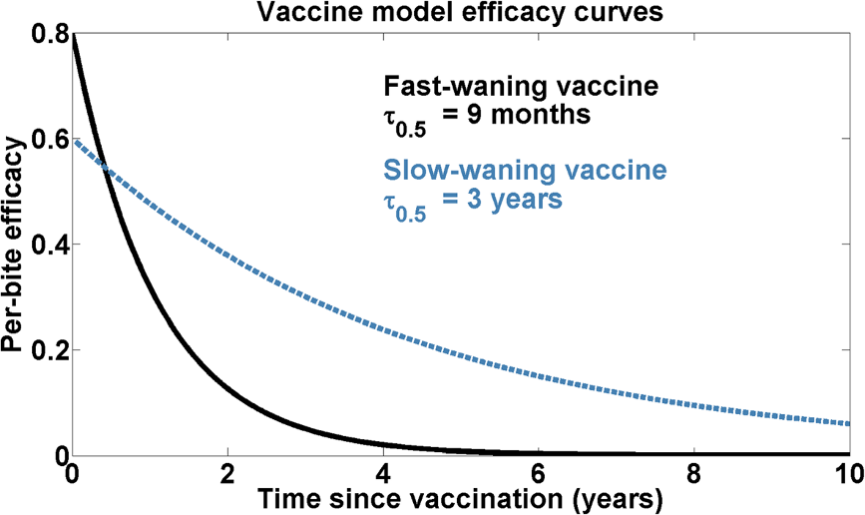
**The two pre-erythrocytic vaccine models evaluated: 80% efficacy with nine-month halflife and 60% efficacy with three-year halflife.**

Each individual simulation tracks a cohort of 1,000 individuals from birth until ten years of age. Individuals receive vaccination between six and nine months of age. This cohort-tracking simulation setup necessarily considers only individual-level protection, ignoring any population-level effects of the vaccination. Crosschecks of results from cohort-based and full-population models indicate that vaccinating only newborns with a waning vaccine produces minimal effects on population-level transmission intensity. The pre-erythrocytic vaccine model is characterized by two parameters: an initial degree of protection against infection and a half-life of protection. The degree of protection is defined as the reduction in probability of successful infection in a single challenge bite. Two vaccine profiles were evaluated against baseline – one with an initial degree of protection of 80% and a half-life of nine months (referred to as fast-waning), and another with an initial protection of 60% but a half-life of three years (slow-waning). Figure 3 illustrates the two vaccine profiles evaluated.

## Results

**Figure 4:**
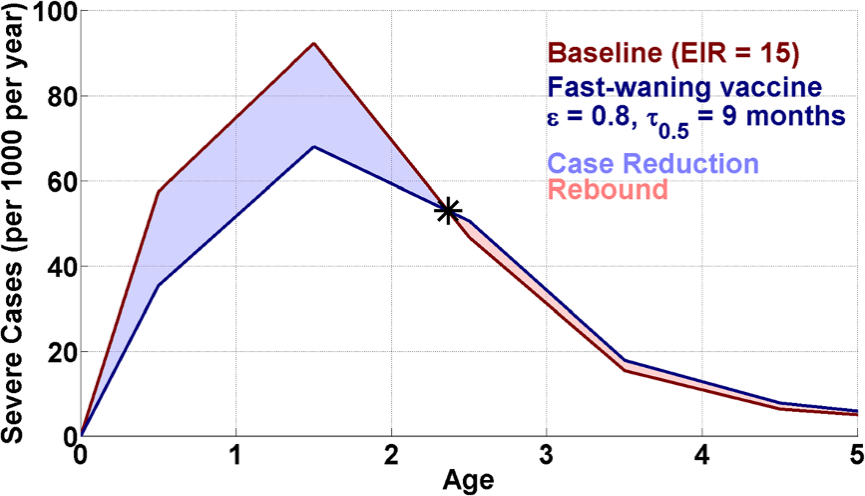
**Severe disease incidence per 1,000 children per year at annual EIR = 15.** The median baseline incidence is in dark red, and incidence with the fast-waning vaccine is shown in dark blue. The region of incidence reduction at young ages is highlighted in light blue. The disease incidence rebounds at ~2.5 years of age, and the rebound is highlighted in light red.

Over essentially the entire range of simulated transmission conditions, the model predicts that a pre-erythrocytic vaccine delivered early in life results in a reduction in the rate of severe malarial incidence for some period of time, followed by a rebound in case counts, when the delayed onset of acquired immunity results in vaccinated children exhibiting a higher rate of severe malaria incidence than unvaccinated children. At all levels of transmission, the median net number of severe cases averted is positive, though it approaches zero at the highest simulated EIR. Figure 4 presents a typical illustrative example of this reduction and rebound behaviour, comparing the median difference in disease incidence in the baseline scenario *vs* the fast-waning vaccine scenario at an annual EIR of 10. The figure shows a strong reduction in disease incidence over the first two years’ post vaccination (at six months of age), with a rebound effect causing a slightly higher rate of severe disease in the vaccinated group as compared to baseline after two-and-half years of age. The relative magnitudes and timescales of case reduction and rebound are most strongly dependent on the magnitude of the EIR and the vaccine profile, and less so on the model input parameters or the seasonality (though it should be noted that the idea of optimizing the timing of vaccination with respect to local seasonal transmission was not tested). This section will thus focus on case reduction as a function of annual EIR and vaccine profile.

**Figure 5:**
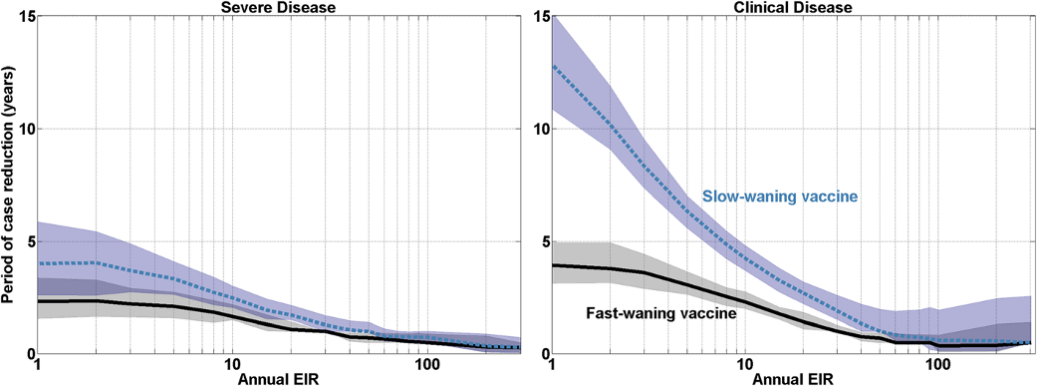
**Period of severe (left) and clinical (right) case reduction prior to rebound for the fast-waning and slow-waning vaccine models.** The lines follow the median output value from the ensemble of model parameters, and shaded areas outline the 68% quantiles.

Figure 5 presents the period of case reduction prior to rebound against the annual EIR for both vaccine models. Referring to Figure 4, the period of case reduction is defined as the interpolated crossing of the incidence curves in baseline and vaccine scenarios (marked in black). The eight stochastic repetitions are averaged, and the median/quantiles are computed over the four seasonal profiles and 170 model parameter sets at each annual EIR value. As should be expected, the protective period decreases as the annual EIR increases for both vaccine profiles, and the slowly waning vaccine provides a substantially longer period of case reduction under low transmission intensity. At higher transmission intensities, there is little difference in the period of case reduction between the two vaccine models. Modelling the vaccine as inducing imperfect, probabilistic protection against infection in a single bite implies that vaccination provides minimal effective protection when exposed to highly frequent infectious biting.

**Figure 6:**
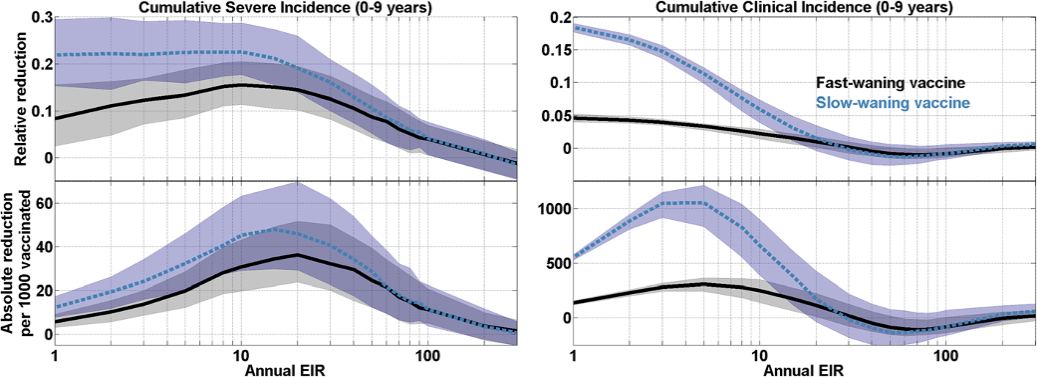
**EIR-dependent reduction in relative (top) and total (bottom) incidence of severe (left) and clinical (right) malaria; incidence, per 1,000 children over the first ten years of life, for both vaccine models.** The lines follow the median output value from the ensemble of model parameters, and shaded areas outline the 68% quantiles.

The fractional (top) and absolute (bottom) reduction in the cumulative severe (left) and clinical (right) incidence over the first ten years of life are presented against annual EIR for both vaccine profiles in Figure 6. The total severe incidence reduction from distribution of a pre-erythrocytic vaccine exhibits a broad maximum over a range of moderate EIR values from roughly 10 to 40. Under mild transmission conditions, the rate of severe malaria is sufficiently low that, although the vaccine is highly efficacious in terms of relative case reduction, the absolute number of cases averted is quite small. And under intense transmission, transmission is sufficiently strong that a vaccine that acts to randomly block a maximum of 60 or 80% of potential acquisitions will provide only a slight delay in disease acquisition, with insignificant net burden reduction. It should be noted that the uncertainties in the effects of vaccination arise from varying the input parameters within the acceptable region defined in calibration; uncertainties due to model structure (e.g., individual- or site-level heterogeneity) are outside of the scope of the present work.

The maximal reduction in cumulative clinical incidence occurs at lower annual EIRs, with a peak around an EIR of 5. At EIRs from 30–100, the rebound effect can actually outweigh the initial case reduction, such that recipients of the vaccine experience slightly increased cumulative rates of clinical incidence. Reductions in transmission intensity have previously been observed to produce increased cumulative clinical incidence, so it is not unexpected that delayed acquisition of protective immunity due to vaccination and increased biting risk at older ages would combine to produce a net increase in cumulative incidence. At the highest EIRs tested, vaccination produces no significant change in total clinical incidence, as the intensity of transmission overwhelms the partially protective vaccination.

**Figure 7:**
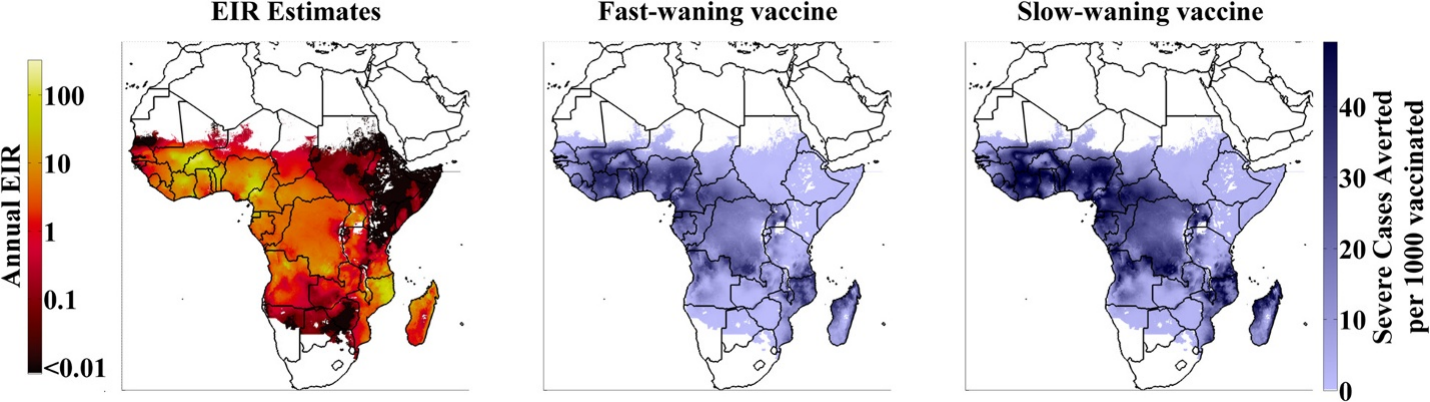
**(left) Malaria Atlas Project estimates of transmission intensity (EIR values interpolated from PfPR**_**2–10**_ **).** Expected cases averted per 1,000 children vaccinated over a ten-year horizon, 100% infant immunization coverage with (center) fast-waning vaccine and (right) slow-waning vaccine.

The Malaria Atlas Project [40] has produced estimates of transmission conditions on a finely partitioned spatial grid throughout sub-Saharan Africa. These estimates are combined with the results of this parameter ensemble evaluation to produce the visualizations in Figure 7. The left panel maps the estimated EIR values, and the center and right panels represent, for the fast and slow-waning vaccines, respectively, the expected number of cases averted per 1,000 children over a ten-year horizon. The resulting maps project where the rollout of a pre-erythrocytic vaccine would produce optimal benefits. The magnitude of the total cases averted assumes 100% vaccination coverage of newborns, and does not consider coordinated rollout of the vaccine with other transmission-reducing interventions.

## Conclusions

Routine immunization of infants has proven to be an incredibly effective tool for reducing the burden of numerous infectious diseases, particularly so for diseases in which protection is long-lasting and naïve individuals dominate the transmission chain. The present work finds that routine immunization with a vaccine providing imperfect protection against acquisition of malaria would provide optimal burden reduction within a range of EIRs from approximately 10–40, where there is sufficient burden for a vaccine to produce large effects, but the frequency of parasite inoculation is not so high as to overwhelm the vaccine’s protective effects. Simulations indicate that, due to malaria’s ability to re-infect human hosts numerous times, infant immunization alone does not strongly perturb population-level transmission dynamics, and efforts towards local elimination must include broader intervention strategies. This evaluation was performed using an ensemble of model parametrizations, and it is found that the results are robust within a broad volume of model parameter space consistent with existing population-level measurements.

Extensions to this work may consider the effects of routine boosters as the vaccine’s protective effects wane, mass vaccinations at sufficient scales to affect malaria’s transmission chain, or correlated distribution of other anti-malarial interventions in regions where the vaccine is deployed. The presented models also do not consider potential differences in overall mortality resulting from shifting a fraction of the severe disease burden onto older children. The calibration framework developed also allows for straightforward addition of new data targets, including similar measurements taken in other regions or different types of measurement (e.g., measured parasite densities), and further work along this direction is also merited.

## Electronic supplementary material

Additional file 1: **Supplemental material: provides further details of calibration setup, results, and validation.** (PDF 2 MB)

## Abbreviations

EIR: Entomological inoculation rate
PfPR_2–10_: Proportion of two- to ten-years-olds testing positive for *Plasmodium falciparum*
PfEMP: *P. falciparum* erythrocyte membrane protein
MSP: Merozoite surface proteins
RBC: Red blood cells.
