# Supplementary material for: Calibration of an intrahost malaria model and parameter ensemble evaluation of a pre-erythrocytic vaccine
Source: Malar J. 2015 Jan 7;14:6. doi: 10.1186/1475-2875-14-6 (PMC4326442; doi:10.1186/1475-2875-14-6)
Supplement: Supplementary file 1 — Additional file 1: Supplemental material: provides further details of calibration setup, results, and validation. (PDF 2 MB) [file 12936_2014_3675_MOESM1_ESM.pdf]

Supplemental material

Details of calibration setup

Because of the interactions between the human and vector systems in the EMOD model, broad variations in human immune dynamics induce variations in the transmission conditions. This presents a difficulty in the calibration process, as the target distributions are age-specific prevalence and incidence curves determined at measured EIRs. It is computationally expensive to adjust vector populations at each tested set of intrahost parameters to compare results at equal transmission levels. To remove this complication, the measured transmission conditions were reproduced by removing vectors from the simulation entirely, and instead subjecting the simulated individuals to periodic sporozoite challenge at a known intensity. The frequency of challenge is varied monthly to mimic seasonal variation in EIR (Table 1 presents these arrays of monthly EIRs). This setup allows for consistent comparison of simulated prevalence and incidence outputs at different sets of intrahost model parameters, without the expense of the "nuisance dimensions" induced by human-vector feedback.

Seasonal variations in EIR were taken from the literature where available. When unavailable, seasonal variation was set through simulation with appropriate climate data, or assumed to be similar to published values for nearby sites; Table 1 lists the sources for each site studied. Both [1] and [2] characterize their study regions primarily using *falciparum* parasite prevalence in 1-9 or 2-10 year olds rather than EIR. Seasonal variations at these sites were assumed to be similar to published measurements in nearby settings. Simulations were then run to characterize the annual average $PfPR_{2\text{-}10}$ as a function of the annual EIR given the assumed seasonal variations. The annual EIR values used in calibration were interpolated to reproduce the

measured $PfPR_{2-10}$ in the data sources. As the target data are averaged on timescales of a year or longer, the seasonality in EIR is expected to be a second-order effect in the calibration compared to the overall magnitude of the EIR.

These two choices of simulation architecture (direct sporozoite challenge vs. vector-mediated transmission, and cohort-tracking rather than population sampling) were made to reduce the runtime of individual simulations and the total number of simulations required for the calibration. Calibrating individual immune system parameters under these conditions does carry the implicit assumptions that both herd effects and human-vector system dynamics are negligible in the context of this calibration, i.e., that the model outputs will be similar in a full population experiencing vector transmission at the appropriate intensity. Tests of these assumptions have indicated that the mean behavior is well preserved, but that full population simulations exhibit increased variance in outputs, most prominently under very low transmission conditions.

When data was taken from a population receiving no or limited antimalarial interventions, the simulations initialize a single population of individuals at age 0, and track prevalence and incidence in this population as they age under repeated malaria exposure. Births are not modeled in this cohort-tracking setup. A different setup is required when age-targeted antimalarial interventions were present, as the immune systems of older individuals evolved in the absence of these interventions. For these simulations, births are enabled, and an age-targeted intervention is distributed after the simulation has run sufficiently long to burn in the adult population immune characteristics. Simulation setups are summarized in Table 1.

Model details – maternal antibodies and severe disease

The EMOD model has multiple means of accounting for maternal antibodies; the most detailed is to assign each birth to an individual mother and base the new infant's maternal antibody protection on the mother's prior exposure. In this cohort-tracking setup, this option is not available; infants are instead assigned a constant level of maternal antibody protection at birth, and the degree of this protection is based on the local transmission conditions. A set of simulations was run after the prevalence/incidence calibration to determine the average fraction of PfEMP1 variants that individuals in the population of potential mothers (defined here as females aged 14-45) have experienced. The results were fit to a rational function, and the final fitted function is:

$$\frac{\mathbf{0.9 * \left(2.5 * 10^{-5}\, \mathit{EIR}^2 + 0.35 * \mathit{EIR}\right)}}{\mathbf{2 + 0.35 * \mathit{EIR}}}$$

The output is plotted in Figure S1. This maternal antibody protection is provided at birth, and decays at a rate of 1% daily (half-life of approximately 10 weeks). Maternal antibodies act in concert with PfEMP1 and nonspecific antibodies to kill infected red blood cells in the model. The calibration of maternal antibody levels calibrates an overall scalar on the total maternal antibody protection; the functional form is not varied in calibration.

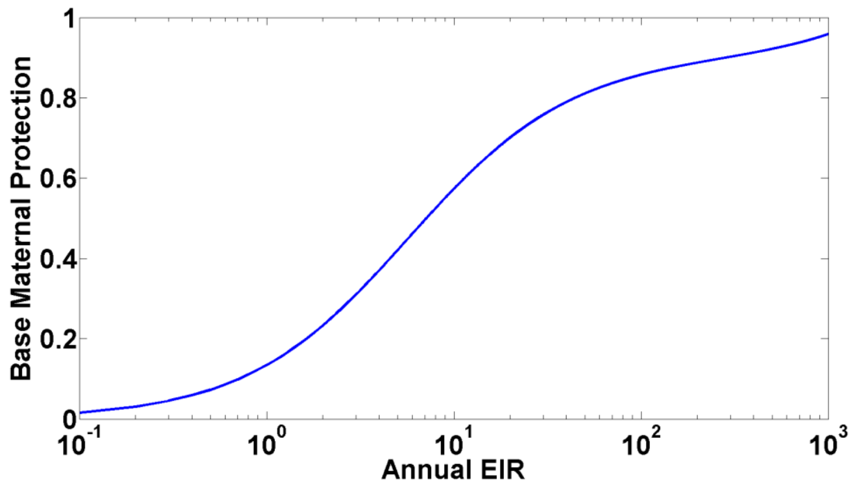

Figure S1: EIR dependence of the average fraction of antigenic variants experienced by potential mothers in the simulated population. This quantity defines the degree of maternal antibody protection provided to infants at the beginning of a simulation, when simulations are run in "cohort-tracking" mode with no births modeled.

The functions translating RBC counts, asexual parasite densities and fever levels to probabilities of diagnosis are sigmoid (logistic) functions characterized by a width and a midpoint. The functional form is:

$$P(x) = \frac{1}{1 + e^{k(1-\frac{x}{x_0})}}$$

Where k represents the inverse width, and x_0 is the threshold, at which the probability is equal to 0.5. Figure S2 presents the behavior of these functions for the fever levels (left) and parasitaemias (right), using 1000 random samples of values for the midpoints and widths from the preferred region post-calibration. The functions are binned in the x-dimension (temperature or parasite density), and the median and 68/95/100% quantiles of the probability at a given x are plotted and shaded in y. In the preferred parameter space post-calibration, the probability of experiencing a severe cerebral malaria episode is essentially zero for low-grade fevers, but increases rapidly as the fever crosses approximately 40.5^{o}C (base body temperature is assumed to be 37°C). Similarly, a severe disease episode caused by hyperparasitaemia is highly unlikely

at parasitaemias below approximately 200,000 per µl, rapidly increasing to a probability of 1 in most samples by approximately 400,000 per µl.

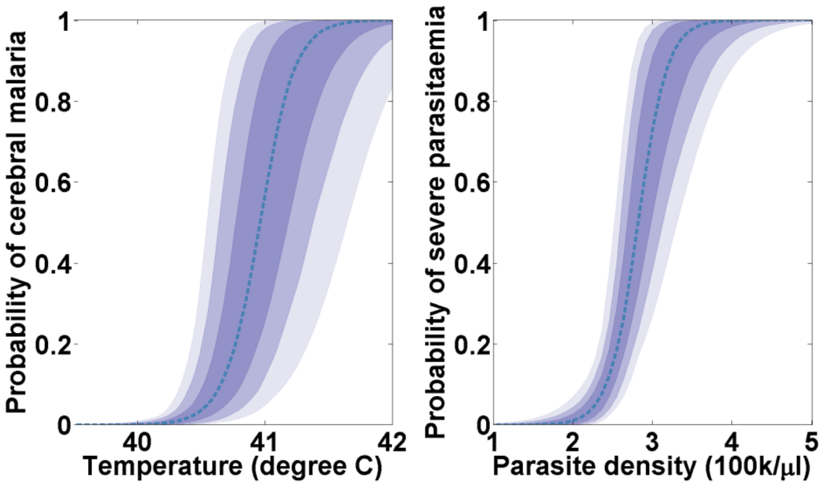

Figure S2: (Left) Sigmoid functions describing the probability of being diagnosed with severe malaria as a function of current body temperature. (Right) Sigmoid functions describing the probability of being diagnosed with severe malaria as a function of current parasitaemia. Sigmoid curves are computed using a random sampling of parameters within the acceptable volume post-calibration. The dashed line represents the median, and the blue-shaded regions represent the 68/95/100% quantiles of the posterior curves (median/quantiles computed within bins in the X dimension).

Likelihood functions

The likelihood of a set of model parameter values θ given the target outcomes *d* is, by definition, the probability of observing the outcome data *d* given the parameter vector θ; that is:

$$\mathcal{L}(\theta|d) = P(d|\theta)$$

Given the complex nature of the model, exact evaluation of the likelihood function is infeasible, and thus approximations to the true likelihood are utilized in the importance sampling algorithm. To perform this approximation, each dataset targeted by the calibration is assumed to follow a simple appropriate distribution – prevalence outcomes are treated as binomial variables, incidence outcomes as Poisson variables. The simulation outcomes are used to constrain the hyperparameters of a corresponding conjugate prior (beta and gamma priors on binomial and

Poisson parameters, respectively). The likelihood of observing the data is then approximated by the posterior predictive distribution describing new observations given the simulated outcomes.

As an illustrative example, the likelihood for a set of calibration parameters $\boldsymbol{\theta}$ given binomial prevalence data $\boldsymbol{d}$ (consisting of $\boldsymbol{k_d}$ parasite-positive observations out of $\boldsymbol{n_d}$ total observations) is approximated as follows. The simulation outputs $\boldsymbol{k_s}$, $\boldsymbol{n_s}$ are used to update an initially uniform beta prior to provide a simulation-informed posterior distribution:

$$Beta(p|1,1) \to Beta(p|1+k_s, 1+n_s-k_s)$$

The likelihood is then approximated using the posterior predictive distribution, computed by marginalizing over the binomial parameter:

$$P(d|\theta) = \int P(d|p)P(p|\theta)dp = \int Bin(k_d|n_d,p)Beta(p|\alpha,\beta)\,dp = \ BetaBin(k_d|n_d,\alpha,\beta)$$

Where $(\alpha,\beta) = (1+k_s, 1+n_s-k_s)$. Approximate likelihood functions for Poisson incidence data with a gamma prior are obtained in a similar fashion. Each age bin in each region is treated as an independent measurement, and the final likelihood is the product of likelihoods over all of the age bins and regions.

Certain assumptions are implied by this definition of the likelihood function. Points in each age bin are not truly independent at a given $\boldsymbol{\theta}$, but this approximate likelihood ignores correlations across age bins. However, treating the age bins as independent is sufficient to obtain generally good fits; the mechanistic nature of the model appears to induce the appropriate correlations in favored regions of parameter space. Also, describing the data using binomial and Poisson distributions presumes a lack of underlying heterogeneity at the individual level (beyond

heterogeneity induced by stochasticity). Investigation of the data on a disaggregated basis may reveal overdispersion due to individual heterogeneity, but this heterogeneity is not investigated here.

Prevalence/incidence calibration

A subset of the available malaria model parameters deemed most likely to exert the dominant effects on the population-level prevalence and incidence measurements were included in calibration, while the remaining parameters were held fixed at values that were calibrated by hand to other data sources. This set of fixed parameters includes all of the antibody level/capacity growth rates, whose effects are much more readily calibrated to time courses of individual infections. The antibody memory levels had been hand-calibrated and were excluded here. Finally the strength of antibody response to the major pfEMP1 variants was calibrated on time courses of parasitemia in naïve individuals and excluded here; the strengths of antibody responses to antigens with less population variance are expected to have larger effects on long-term immunity after repeated exposure and are included. The final set of included parameters are the number of antigenic variants in each of the three immune compartments, the strengths of antibodies against the MSP1 antigens and shared minor epitopes, and switching rate between PfEMP1 antigenic variants in a single infection.

A summary of the results of the calibration exercise are presented in Figure S3. The calibration was performed over 6 dimensions, and show the log-likelihood values (color) collapsed onto 3 different 2-dimensional spaces (# of PfEMP1 variants and switching rate, # of MSP1 variants and MSP1 antigenicity, # of nonspecific types and nonspecific antigenicity). The log-likelihood values are truncated at the low end, to prevent excessive compression of the color

scale in the region of high likelihood. The parameter sets that pass the acceptance threshold are highlighted in red, and the convex hull enclosing all of these parameter sets is outlined in black dashed lines. The increased sampling near the highest likelihood regions is a desired consequence of the iterative IMIS calibration algorithm. Because 4 dimensions are collapsed for the images, this convex hull in each 2-d slice also encloses a number of poorly-matching simulations; these generally indicate poor parameter values in one or more of the other dimensions. Collapsing the 6D space onto two dimensions for presentation hides covariances in the other dimensions, but the 2D planes shown here represent the dominant interactions between the parameters.

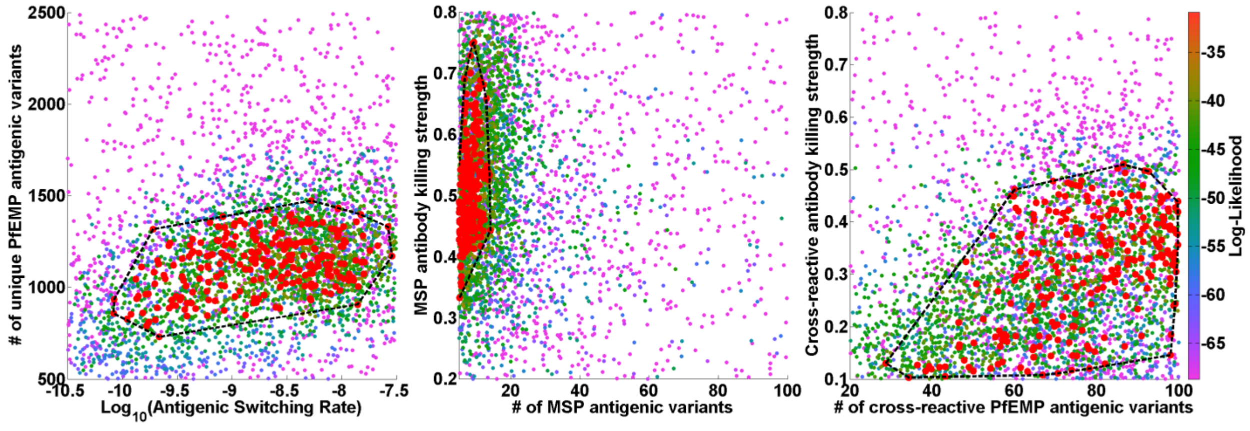

Figure S3: Summary of results of prevalence/clinical incidence calibration. The sampled points, collapsed onto 2 dimensional spaces are shown and colored by log-likelihood. Points passing the log-likelihood threshold are highlighted in red, and the projection of the convex hull onto the 2-D space is outlines in black.

The calibrated region of unique PfEMP variants and antigenic switching rate contains the values of these parameters that have been preferred in previous work [3, 4], though the allowed range of antigenic switching rates is rather broad, and previous work on infection durations provides stronger constraints. The contrast between the MSP and cross-reactive immune compartments is also interesting; the preferred region restricts the MSP compartment to have few

variants that are effectively targeted by the antibody response, while the cross-reactive compartment is allowed a larger number of variants with a relatively weak antibody response.

Severe disease calibration

The classification of a severe malaria incident in the model is complicated by the plurality of potential manifestations of malaria that can result in a diagnosis of severe malaria. The likelihood function employed predominantly targets the absolute incidence of severe disease, but also features a couple of Gaussian penalty terms that aim to keep the fractions of anemic, cerebral, and other severe disease near the observed values.

Because the cohort is initialized, rather than born to specific mothers, maternal antibodies are not directly transmitted in these simulations. To mimic the effect of maternal antibodies, an EIR-dependent initial immunity is conferred to the cohort on day 0, and calibration is performed on the overall scaling of this immunity.

Results are presented in Figure S4 in a fashion similar to the results of the prevalence/incidence calibration. The three figures present scatter plots of the log-likelihood collapsed onto 2 dimensions, with the figures in the left column showing all simulated points on a color scale; parameter sets passing the acceptance threshold are outlined in red, and a black dashed line depicts the 2-D convex hull of all acceptable parameter sets. The convex hull does not do a good job capturing the somewhat concave relationship between the threshold and

inverse width of the parasitaemia sigmoid parameters. Importance sampling can be used within the convex hull to ameliorate this issue.

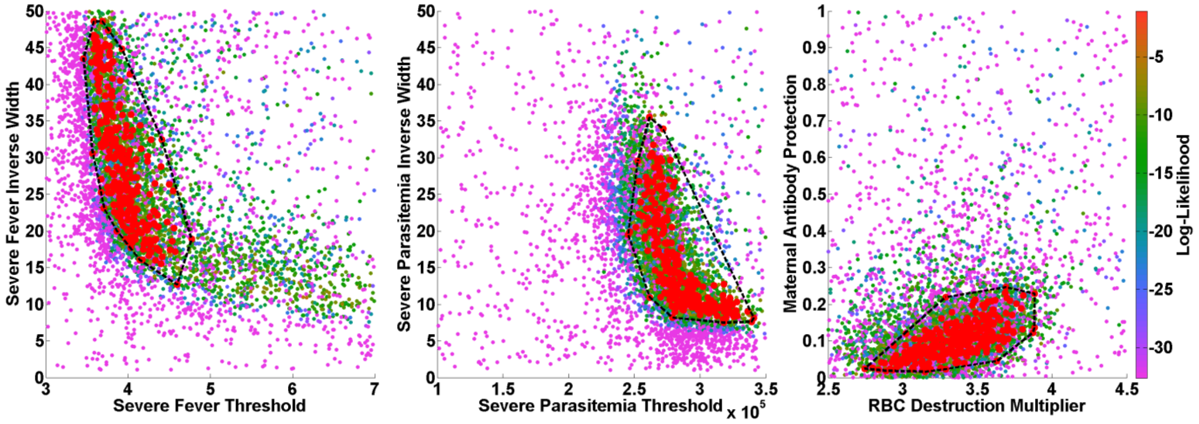

Figure S4: Summary of results of severe disease incidence calibration. The sampled points, collapsed onto 2 dimensional spaces are shown and colored by log-likelihood. Points passing the log-likelihood threshold are highlighted in red, and the projection of the convex hull onto the 2-D space is outlines in black.

Out-of-sample validation of calibration results

The one-hundred and seventy model parameter samples used to evaluate vaccine efficacy were also utilized to check the performance of the calibration results against out-of-sample data. Only the baseline (no-vaccine) runs of the simulation were included; runs in which vaccine distribution was simulated are obviously unsuitable for validation purposes. Data were obtained from three sources [5][6][7], which each treat a dataset compiled from a number of sources.

Figure S5 presents a comparison of data and calibrated model predictions of $Pf\mathrm{PR}_{<15}$ vs. annual EIR. The data points are obtained from Figure 1a of [5] and shown with the best model fit presented in that work. The data and fit extend to annual EIR values both below and above those simulated in the present work. Within the bounds of EIRs treated in this work (1-300), the median model outputs are not in disagreement with the observed data. However, the uncertainty

on the calibrated model outputs, particularly at annual EIRs below 10, does not capture the degree of variation observed in the data. This result is not unexpected; the model treats the population as homogenous in both immune system dynamics and acquisition risk. The data are drawn from a number of underlying studies, which also introduces additional heterogeneities; as noted by the authors of [5], different studies used different age ranges to define *Pf*PR and different methods of estimating the annual EIR.

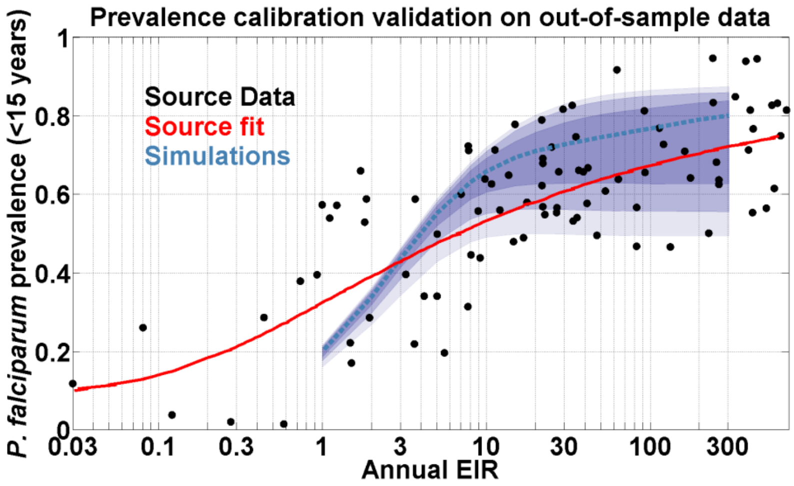

Figure S5: Comparison of model predictions of *Pf*PR in children under 15 vs. EIR, against data outside of the calibration sample. The dashed blue line indicates the median model prediction, while the shaded regions present 68/95/100% quantiles of the simulation outputs. The black points represent data from a number of field studies, compiled together in [5]. The red line indicates the best fit model proposed by the authors.

Figure S6 presents the calibrated model predictions for the age profile of clinical incidence under six different broadly defined transmission categories, as presented in [6], along with the best fits presented in Figure 2 of that work. The three columns represent low (EIR<10), moderate (10<EIR<100), and high (EIR>100) transmission intensities, and the two rows separate low and high seasonality in transmission (where high seasonality, or “marked seasonality” in the source paper, is defined as ≥75% of episodes being concentrated in ≤6 months of the year). The absolute incidence rates are normalized away, so that all curves sum to 100%. As EIR increases, the model qualitatively captures the increase in the fraction of incidents experienced early in life,

though not quite to the degree of the fits. The effect of seasonality is also not as marked in the model as in the fits; the calibrations were performed with data aggregated on 1-year age bins, which may smooth away the effects of seasonality in calibration. Modelling seasonal variations in incidence is a target of ongoing calibration work.

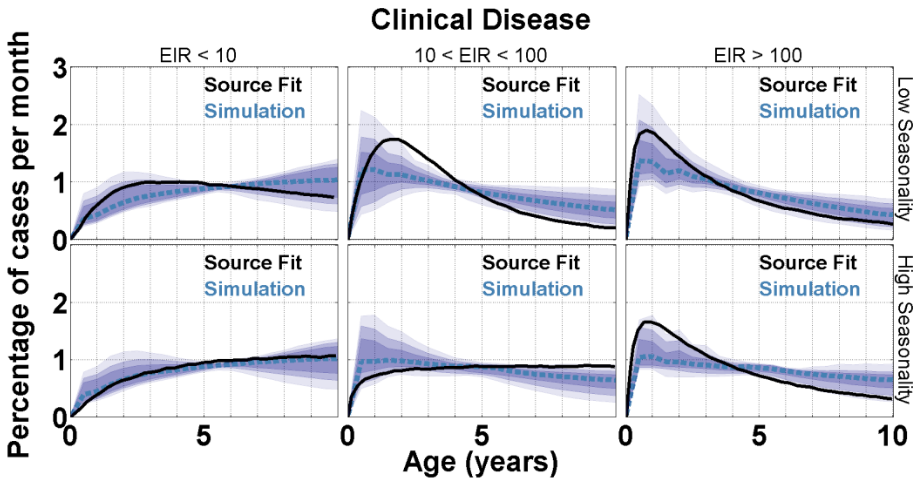

Figure S6: Model predictions of the age profile of clinical incidence in children under 10, along with the best fits obtained in [6]. The dashed blue line indicates the median model prediction, while the shaded regions present 68/95/100% quantiles of the simulation outputs; the black lines represent the best fits presented in the source paper. The absolute incidence rate is normalized out, so that all curves sum to 100%.

Figure S7 presents the calibrated model predictions for the age profile of severe malaria incidence from the model against hospital admission rates presented in [6], along with the best fits presented in that work. The 6 panels are analogous to those in Figure S6, and absolute rates are again normalized out. The low-intensity, low-seasonality panel does not contain a fit for hospital admission rates in the source paper, but the model results are included for completeness. The model predictions exhibit good agreement with the fits in [6], with the exception of the high-

seasonality, moderate-intensity panel; the fit in the source indicates that increased seasonality shifts the burden to older age bins, and this effect is not present in the model outputs.

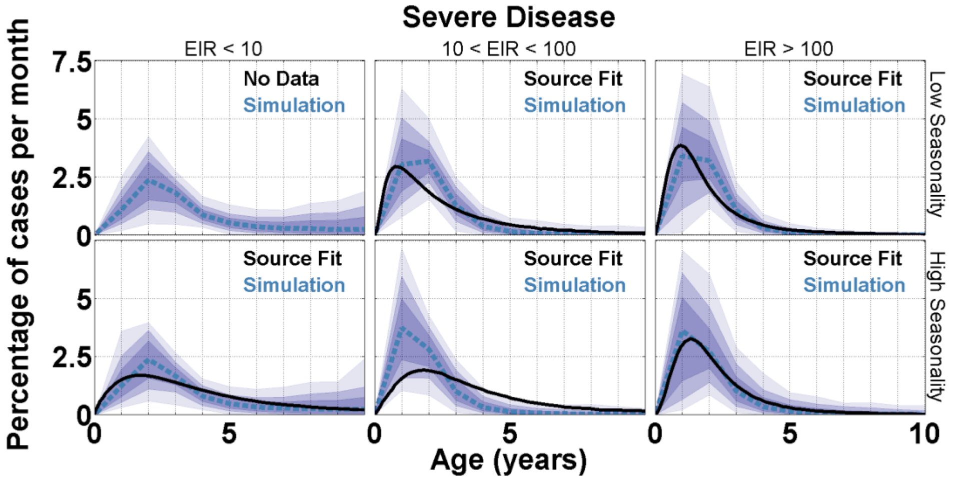

Figure S7: Model predictions of the age profile of severe incidence in children under 10, along with the best fits obtained in [6]. The dashed blue line indicates the median model prediction, while the shaded regions present 68/95/100% quantiles of the simulation outputs; the black lines represent the best fits presented in the source paper. The absolute incidence rate is normalized out, so that all curves sum to 100%.

Finally, Figure S8 presents model predictions of the all-age incidence (per 1000 people per year) vs. *Pf*$PR_{2\text{-}10}$ as presented in [7] (Figure 6 in that work). The data extends to prevalence levels lower than those simulated in this work, but the model and data are not in disagreement at the prevalence levels available in both. The median model prediction is higher than the median of the posterior distribution of the prevalence-incidence relationship obtained in [7]. A potential reason for this disagreement is that, as noted in the main text, the simulations performed for the vaccine evaluation do not track individuals for their entire lifetime; as incidence rates tend to decline in older age groups, the presented incidence rates are likely biased upward from what full-lifetime simulations would predict. Furthermore, as mentioned in the main text, the studies used for calibration employed active case detection 3 times per week, and higher frequency of case detection visits has been shown to produce higher estimates of incidence [8, 9]; this effect may also bias the present calibration towards higher incidence rates.

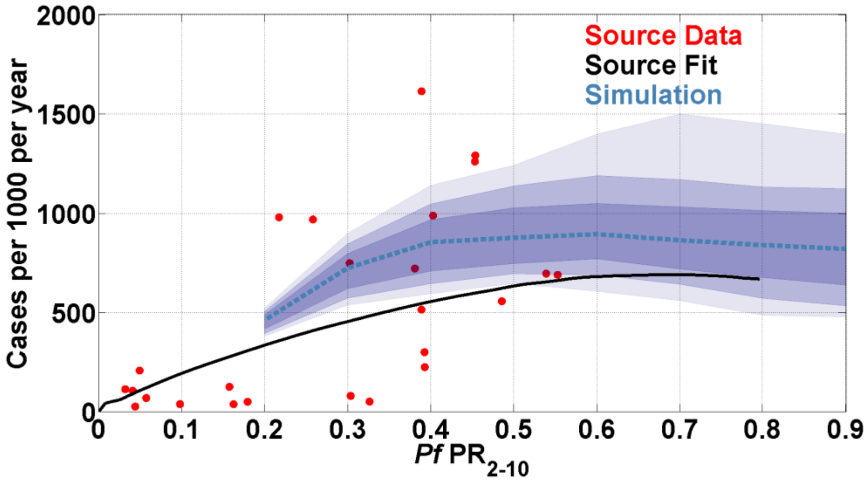

Figure S8: Model predictions of the all-age incidence (per 1000 people per year) vs. *Pf*PR$_{2\text{-}10}$ as presented in [7]. The dashed blue line indicates the median model prediction, while the shaded regions present 68/95/100% quantiles of the simulation outputs. The median of the posterior distribution obtained in [7] is presented in black, with data shown as red points.

Model parameter sampling

To evaluate model dependence on the effects of a pre-erythrocytic vaccine, 170 models are resampled using the results of the calibration procedure. Both of the 6-dimensional calibration steps are finalized by defining a set of "acceptable" points using an application of Wilk's theorem, taking the maximal likelihood to be the null hypothesis:

$$2 \log \mathcal{L}(\vec{x}) > 2 \max(\log \mathcal{L}(\{\vec{x}\})) - \; F^{-1}(0.9)$$

Where $F^{-1}(k, x)$ is the inverse CDF of the χ^2 distribution with k degrees of freedom. The $\vec{x}$ satisfying this criterion are then used to construct a convex hull, and the enclosed parameter space volume is considered to be the set of well-calibrated parameter sets. Being defined by a limited number of points in a high-dimensional space, the hull almost certainly encloses parameter sets that would fail the above criterion – this can be ameliorated by employing importance sampling from points within the volume. However, the ensemble model vaccine

evaluation employs uniform sampling of the interior of the convex hull, to provide a broader exploration of predicted vaccine performance within this parameter space volume.

Table 1: Description of simulation setups for calibration

**Region**	**Monthly EIRs**	**Source for EIR**	**Interventions**	**Population**
Matsari (prevalence)	68 *[0.02, 0.05, 0.10, 0.15, 0.21, 0.20, 0.16, 0.08, 0.02, 0.00, 0.00, 0.01]	Simulation	None	Cohort of 100
Namawala (prevalence)	329* [0.13, 0.21, 0.08, 0.14, 0.15, 0.08, 0.04, 0.03, 0.03, 0.01, 0.04, 0.05]	[10]	None	Same as above
Rafin Marke (prevalence)	18*[0.03, 0.06, 0.09, 0.14, 0.18, 0.18, 0.15, 0.10, 0.03, 0.01, 0.01, 0.02]	Simulation	None	Same as above
Sugungum (prevalence)	132*[0.02, 0.05, 0.10, 0.15, 0.21, 0.20, 0.16, 0.08, 0.02, 0.00, 0.00, 0.01]	Simulation	None	Same as above
Dielmo (incidence)	200*[0.07, 0.08, 0.04, 0.02, 0.03, 0.04, 0.22, 0.13, 0.18, 0.09, 0.07, 0.05]	[11]	Upon fever, children <10 have a 30% daily probability of receiving a full course of quinine	Absolute birth rate of 10 per year; interventions begin at year 40.
Ndiop (incidence)	20*[0.02, 0.01, 0.04, 0.00, 0.00, 0.00, 0.32, 0.11, 0.24, 0.20, 0.04, 0.03]	Simulation	Same as Dielmo	Same as Dielmo

Bakau	.11 * [0.02, 0.01, 0.04, 0.00, 0.00, 0.00, 0.32, 0.11, 0.24, 0.20, 0.04, 0.03]	Seasonality assumed to be Ndiop-like, magnitude interpolated	None	Cohort of 10000, followed from birth.
Sukuta	3 * [0.07, 0.08, 0.04, 0.02, 0.03, 0.04, 0.22, 0.13, 0.18, 0.09, 0.07, 0.05]	Seasonality assumed to be Dielmo-like, magnitude interpolated	None	Same as above
Kilifi – North	5.6 * [0.11, 0.04, 0.00, 0.08, 0.01, 0.30, 0.31, 0.09, 0.01, 0.00, 0.02, 0.03]	Seasonality from [12], magnitude interpolated	None	Same as above
Kilifi – South	34.1 * [0.11, 0.04, 0.00, 0.08, 0.01, 0.30, 0.31, 0.09, 0.01, 0.00, 0.02, 0.03]	Seasonality from [12], magnitude interpolated	None	Cohort of 2000

Siaya	55 * [0.13, 0.21, 0.08, 0.14, 0.15, 0.08, 0.04, 0.03, 0.03, 0.01, 0.04, 0.05]	Seasonality assumed to be Namawala-like, magnitude interpolated	None	Cohort of 2000

1. Okiro EA, Al-Taiar A, Reyburn H, Idro R, Berkley JA, Snow RW: **Age patterns of severe paediatric malaria and their relationship to Plasmodium falciparum transmission intensity**. *Malar J* 2009, **8**:4.

2. Snow RW, Omumbo JA, Lowe B, Molyneux CS, Obiero JO, Palmer A, Weber MW, Pinder M, Nahlen B, Obonyo C, Newbold C, Gupta S, Marsh K: **Relation between severe malaria morbidity in children and level of Plasmodium falciparum transmission in Africa**. *Lancet* 1997, **349**:1650–1654.

3. Eckhoff PA: **Malaria parasite diversity and transmission intensity affect development of parasitological immunity in a mathematical model**. *Malar J* 2012, **11**:419.

4. Eckhoff P: **P. falciparum Infection Durations and Infectiousness Are Shaped by Antigenic Variation and Innate and Adaptive Host Immunity in a Mathematical Model**. *PLoS ONE* 2012, **7**:e44950.

5. Smith DL, Dushoff J, Snow RW, Hay SI: **The entomological inoculation rate and Plasmodium falciparum infection in African children**. *Nature* 2005, **438**:492–495.

6. Carneiro I, Roca-Feltrer A, Griffin JT, Smith L, Tanner M, Schellenberg JA, Greenwood B, Schellenberg D: **Age-Patterns of Malaria Vary with Severity, Transmission Intensity and Seasonality in Sub-Saharan Africa: A Systematic Review and Pooled Analysis**. *PLoS ONE* 2010, **5**:e8988.

7. Hay SI, Okiro EA, Gething PW, Patil AP, Tatem AJ, Guerra CA, Snow RW: **Estimating the Global Clinical Burden of Plasmodium falciparum Malaria in 2007**. *PLoS Med* 2010, **7**:e1000290.

8. Snow RW, Craig M, Deichmann U, Marsh K: **Estimating mortality, morbidity and disability due to malaria among Africa's non-pregnant population.** *Bull World Health Organ* 1999, **77**:624–640.

9. Patil AP, Okiro EA, Gething PW, Guerra CA, Sharma SK, Snow RW, Hay SI: **Defining the relationship between Plasmodium falciparum parasite rate and clinical disease: statistical models for disease burden estimation**. *Malar J* 2009, **8**:186.

10. Smith T, Charlwood JD, Kihonda J, Mwankusye S, Billingsley P, Meuwissen J, Lyimo E, Takken W, Teuscher T, Tanner M: **Absence of seasonal variation in malaria parasitaemia in an area of intense seasonal transmission**. *Acta Trop* 1993, **54**:55–72.

11. Rogier C, Tall A, Diagne N, Fontenille D, Spiegel A, Trape JF: **Plasmodium falciparum clinical malaria: lessons from longitudinal studies in Senegal**. *Parassitologia* 1999, **41**:255–259.

12. Mbogo CN, Snow RW, Khamala CP, Kabiru EW, Ouma JH, Githure JI, Marsh K, Beier JC: **Relationships between Plasmodium falciparum transmission by vector populations and the incidence of severe disease at nine sites on the Kenyan coast**. *Am J Trop Med Hyg* 1995, **52**:201–206.
